# Interactive effects of systemic inflammation and nutritional status on 1-year functional outcomes in acute ischaemic stroke: a multidimensional biomarker cohort study

**DOI:** 10.3389/fnut.2026.1969226

**Published:** 2026-10-02

**Authors:** Xing-Yu Zhu, Tian-Tian Jiang, Yan-Wei Yin, Guo-Liang Yuan

**Affiliations:** 1 Department of Cardiovascular Medicine, Shuyang Hospital of Traditional Chinese Medicine, ShuYang, JiangSu, China; 2 Neurology, Air Force Medical Center, Chinese People's Liberation Army, Beijing, China

**Keywords:** acute ischaemic stroke, additive interaction, biomarkers, functional outcome, nutritional status, systemic inflammation

## Abstract

**Background:**

Inflammatory cascades and the disruption of protein homeostasis, characterised by diminished serum albumin concentrations, play a pivotal role in the pathophysiological progression of acute ischaemic stroke (AIS). However, previous comparisons across various composite biomarkers have frequently overlooked the mathematical coupling bias introduced by shared components. Furthermore, a quantitative understanding of their non-linear dose–response relationships and synergistic interactions remains elusive. The present study sought to deconstruct highly collinear prognostic indices into distinct pathophysiological axes, delineate their non-linear dose–response trajectories, and quantify the cumulative risk associations and potential interactive effects of joint exposure to systemic inflammation and diminished serum albumin concentrations on 1-year functional outcomes in patients with AIS.

**Methods:**

This retrospective cohort study consecutively enrolled patients with AIS between 2021 and 2023. To circumvent mathematical coupling bias, nine composite indices were rigorously deconstructed into four independent pathophysiological axes. The primary endpoint was a poor functional outcome (modified Rankin Scale score >2) at 1 year after stroke onset. Representative biomarkers were selected using bootstrap-based stability inference. Restricted cubic splines (RCS) and modified Poisson regression with robust standard errors were used to assess non-linear trajectories and synergistic interactions, respectively. A final joint predictive model was constructed following dimensionality reduction via the bootstrap-LASSO algorithm, and the *E*-value was introduced to quantify the robustness of the findings against unmeasured confounding.

**Results:**

We included 495 patients, among whom 111 (22.4%) experienced a poor functional outcome. Following rigorous validation procedures, the neutrophil-to-lymphocyte ratio (NLR), C-reactive protein-to-albumin ratio (CAR), albumin (ALB), and prognostic nutritional index (PNI) were identified as the optimal representative indices for their respective axes. RCS analysis revealed significant non-linear dynamics: the risk trajectory for high-sensitivity C-reactive protein (hsCRP) exhibited a plateau effect, whereas CAR demonstrated an inverted U-shaped pattern. Cross-classification analysis demonstrated that the co-occurrence of elevated NLR and diminished ALB significantly increased the risk of adverse outcomes (adjusted RR 2.45, 95% CI 1.33–4.49), with an absolute risk of 36.9% in the joint exposure group (absolute risk difference [RD] 25.4% versus the reference cohort). Although neither multiplicative nor additive interaction tests achieved statistical significance (multiplicative interaction *p* = 0.856; RERI 0.432, 95% CI spanning zero), this association suggests a potential trend towards compounded high-risk exposure. The bootstrap-LASSO joint predictive model demonstrated excellent calibration (slope 0.805) and clinical net benefit. *E*-value analysis (2.16) and probabilistic simulations corroborated that these findings were highly robust against unmeasured confounders, such as baseline neurological deficits (e.g., missing National Institutes of Health Stroke Scale scores).

**Conclusion:**

The concurrence of systemic inflammatory activation and relatively diminished albumin concentrations constitutes a clinical phenotype robustly associated with long-term adverse outcomes in AIS. By uncoupling collinear biomarkers, this study unmasks complex, non-linear prognostic trajectories that are typically obscured under conventional single-marker or linear assumptions. Furthermore, the compounded high-risk profile of elevated inflammation and diminished albumin provides preliminary epidemiological rationale to inform future investigations into acute-phase anti-inflammatory therapies combined with metabolic homeostasis support.

## Introduction

Acute ischaemic stroke (AIS) is the leading cause of acquired long-term disability worldwide, yet the precise prediction of long-term functional outcomes remains a formidable clinical challenge (1). Although traditional clinical assessment tools (e.g., the National Institutes of Health Stroke Scale [NIHSS]) effectively quantify acute neurological deficits, they frequently fail to capture the underlying systemic immune and metabolic shifts that dictate patients’ long-term recovery trajectories. During the pathophysiological evolution of ischaemic brain injury, disruption of the blood–brain barrier triggers a profound systemic immunoinflammatory cascade; concurrently, stroke-induced sympathetic activation and systemic stress induce an acute hypermetabolic state, driving a negative acute-phase response characterised by a precipitous decline in serum albumin concentrations (2). Emerging evidence indicates that systemic inflammation and the precipitous decline in circulating albumin concentrations are not merely parallel epiphenomena; rather, they propagate a vicious cycle through complex neuro-immune-metabolic cascades, constituting a critical pathophysiological substrate that drives adverse stroke outcomes (3).

In recent years, to integrate prognostic information from both inflammatory and nutritional dimensions, researchers have derived numerous composite indices based on routine haematological tests (e.g., the neutrophil-to-lymphocyte ratio (NLR), C-reactive protein-to-albumin ratio (CAR), and prognostic nutritional index (PNI)), which have shown promise in stroke risk stratification (4). However, the competitive evaluation of these composite indices in cross-sectional comparative studies is hindered by previously underappreciated methodological challenges. Given that these derived indices heavily rely on shared algebraic components (such as lymphocyte counts or serum albumin), incorporating them into the same statistical framework not only induces severe multicollinearity but also inevitably introduces mathematical coupling bias (5). Such spurious correlations, arising from overlapping formulaic structures, might artificially inflate the independent prognostic value of specific biomarkers, thereby misleading the clinical identification of genuine therapeutic targets.

Beyond the structural overlap of these biomarkers, the statistical frameworks previously used to evaluate them possess inherent limitations. Previous studies have predominantly relied on simplistic linear assumptions, thereby neglecting the non-linear dynamic characteristics inherent to biological systems (e.g., threshold effects or plateau phases) (6). Equally crucial is the assessment of synergistic effects: although it is widely postulated clinically that the superimposition of a high inflammatory burden and poor nutritional status significantly exacerbates stroke prognosis, existing literature has largely been confined to the exploration of multiplicative interaction terms. Scant research has utilised rigorous epidemiological models to quantitatively evaluate the true effect of this “double hit” on an additive scale (7). Compared with multiplicative interaction, additive interaction holds substantially greater public health relevance for assessing real-world biological synergies, calculating absolute excess risk, and guiding targeted clinical interventions (8).

Prompted by these methodological considerations and clinical imperatives, this study was designed with four progressive objectives: (1) to deconstruct redundant prognostic indices that share algebraic components into mutually independent core predictive dimensions, thereby circumventing mathematical coupling bias; (2) to delineate the non-linear dose–response trajectories of these isolated biomarkers in relation to 1-year poor functional outcomes after AIS onset using RCS; (3) to explore and quantitatively assess the joint exposure effects and potential additive interactions between systemic inflammation and diminished albumin status; and (4) to develop and internally validate a parsimonious risk prediction model driven by robust feature selection techniques, aiming to provide exploratory clinical insights for early individualised risk stratification and the integrated management of immune dysregulation and protein metabolic dyshomeostasis in patients with AIS.

## Methods

### Study design and participants

This single-centre, retrospective, observational cohort study consecutively enrolled patients with AIS admitted to the Department of Neurology at the Air Force Medical Center, PLA (Beijing, China) between January 2021 and May 2023. The study protocol and the application for a waiver of written informed consent were formally approved by the Institutional Ethics Review Board of the Air Force Medical Center, People’s Liberation Army (PLA). The waiver of informed consent was granted on the grounds that this study solely involved a retrospective analysis of routinely collected clinical data, and all data were rigorously anonymised and de-identified prior to analysis. The study design and reporting strictly adhered to the Strengthening the Reporting of Observational Studies in Epidemiology (STROBE) guidelines and the Transparent Reporting of a multivariable prediction model for Individual Prognosis or Diagnosis (TRIPOD) statement (9).

The inclusion criteria were: (1) age ≥ 60 years; (2) diagnosis of AIS according to the Chinese Guidelines for Diagnosis and Treatment of Acute Ischemic Stroke 2018 (10), confirmed by cranial CT or MRI; and (3) complete clinical and 1-year follow-up data. Exclusion criteria encompassed: missing core laboratory data within 24 h of admission; severe functional dependence prior to stroke onset (modified Rankin Scale [mRS] score ≥ 2); concomitant acute head trauma, severe cardiopulmonary insufficiency, active malignancy, haematological disorders, or neurodegenerative diseases (e.g., dementia); and severe hepatic or renal dysfunction (defined as alanine aminotransferase or aspartate aminotransferase >3 times the upper limit of normal, or serum creatinine >442 μmol/L) (Figure 1).

**Figure 1:**
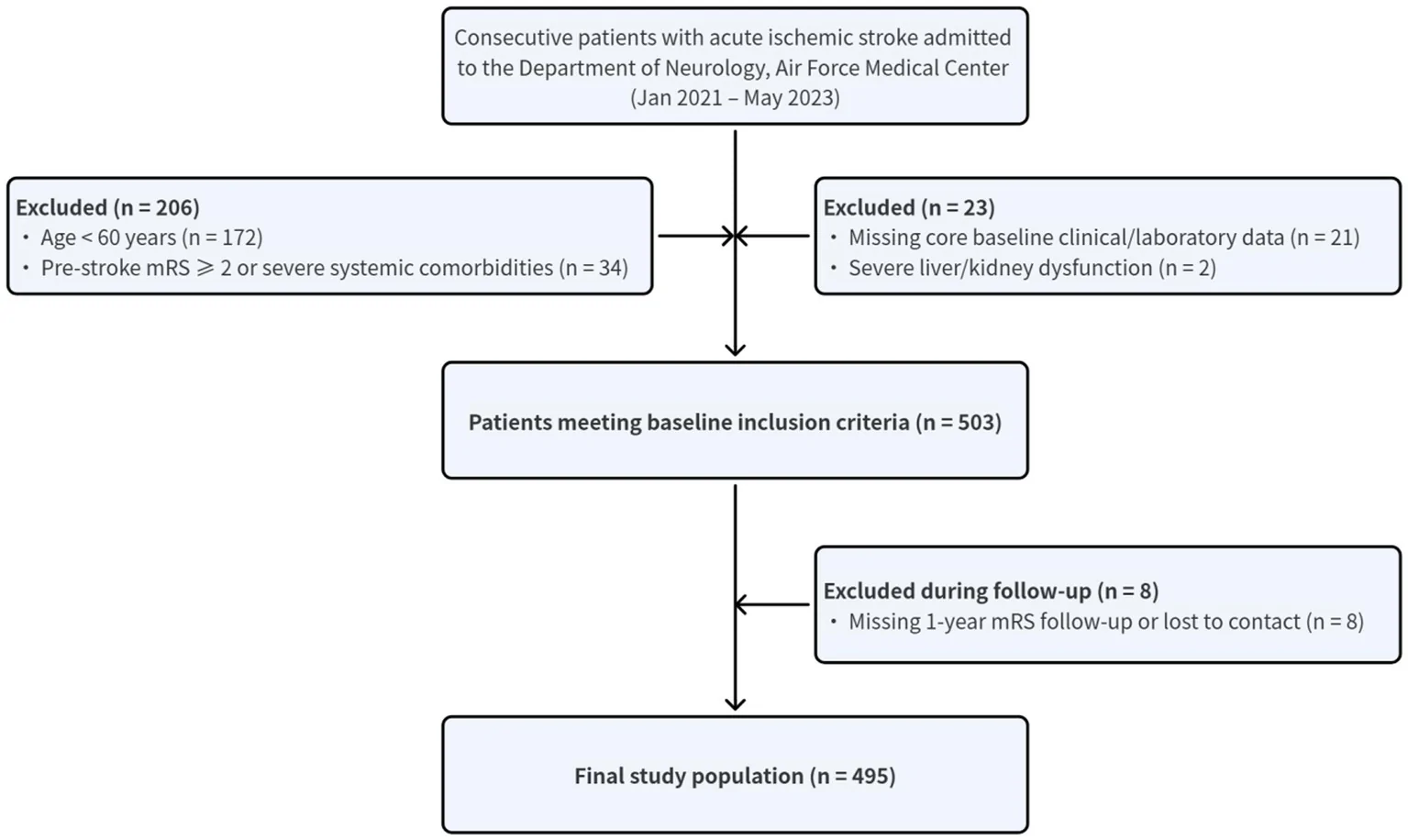
Study flowchart.

### Data collection and quality control

Baseline clinical data were extracted from the electronic medical record system by independent, uniformly trained investigators in a double-blind manner, with third-party cross-checking completed prior to unblinding the follow-up outcomes. The extracted variables encompassed demographic characteristics, medical history, vital signs on admission, and initial fasting peripheral blood laboratory parameters obtained within 24 h of symptom onset (all samples were collected prior to the administration of large-volume intravenous fluid resuscitation, osmotic dehydration, or clinical enteral/parenteral nutritional support, to minimise the interference of haemodilution or therapeutic nutritional supplementation on baseline biomarker concentrations). Logical constraints based on physiologically plausible intervals were pre-set for all biochemical markers; data falling outside reasonable ranges (e.g., zero denominators or exceeding physiological extremes) were traced and verified, and for suspected duplicate entries, only the data from the first admission were strictly retained.

### Biomarker definitions and pathophysiological grouping

Previous studies have often directly compared indices containing shared components (e.g., both containing albumin or lymphocytes). To minimise mathematical coupling bias and multicollinearity, we strictly categorised nine core prognostic indices into four independent pathophysiological axes based on their intrinsic biological mechanisms (11). All derived indices were calculated from the first fasting blood test within 24 h of admission. The specific formulas and units are as follows:

1. Pure inflammatory cell axis (G1): comprising the neutrophil-to-lymphocyte ratio (NLR), systemic immune-inflammation index (SII), and high-sensitivity C-reactive protein (hsCRP). hsCRP was used as the original measured value (mg/L). The formulas for derived indices (cell counts in 10^9^/L) were: NLR = neutrophils/lymphocytes; SII = (platelets × neutrophils)/lymphocytes.
2. Composite inflammatory-nutritional axis (G2): simultaneously capturing acute-phase inflammatory activation and baseline nutritional depletion. The formulas were: CAR = hsCRP (mg/L)/albumin (g/L); FAR = fibrinogen (g/L)/albumin (g/L).
3. Pure nutritional and metabolic axis (G3): comprising albumin (ALB, g/L), glycated hemoglobin (HbA1c, %), and the comprehensive cholesterol-glucose index (CHG). To reflect the patient’s glucolipid metabolic burden with a single continuous parameter, CHG was defined as: CHG = ln[(total cholesterol × fasting blood glucose)/(2 × high-density lipoprotein cholesterol)] (all lipid and glucose units in mmol/L).
4. Comprehensive immune-nutritional axis (G4): represented by the prognostic nutritional index (PNI). Because its components bridge both G1 and G2, it was established as an independent exploratory analysis group. The formula was: PNI = albumin (g/L) + 5 × lymphocytes (10^9^/L).

### Outcomes and covariates

The primary endpoint was an unfavourable functional outcome at 1 year after symptom onset, defined as a modified Rankin Scale (mRS) score of greater than 2; patients who died during follow-up (mRS score 6) were directly assigned to the unfavourable outcome group. Because the events-per-variable ratio constrained by the cohort sample size precluded the implementation of high-dimensional complex models, risk prediction modelling was strictly confined to this binary endpoint. Outcome data were ascertained via structured outpatient evaluations or standardized telephone follow-up.

To control for potential confounding, multivariable analyses adjusted for 11 core clinical covariates: age, sex, hypertension, diabetes, smoking history, admission systolic blood pressure, fasting blood glucose, serum creatinine, and, to adjust for baseline nutritional or haematological biases, body mass index, hemoglobin, and log-transformed D-dimer.

### Statistical analysis

#### Data processing and multiple imputation

Baseline characteristics were analyzed descriptively according to outcome status (favourable vs. poor). The presentation of continuous variables was determined by the Shapiro–Wilk test and skewness coefficients. Pre-set physiological boundaries were used during data cleaning to verify plausibility. For missing data, a complete-case analysis was used for variables with <5% missingness. For variables with 5–30% missingness, following rejection of the missing completely at random assumption by Little’s MCAR test (*p* < 0.05) and further validation of a missing at random mechanism via univariate logistic regression, multiple imputation by chained equations (MICE) was employed to generate 20 complete datasets. Predictive mean matching was mandatorily applied to continuous variables to preserve physiological distributions, and effect estimates were pooled using Rubin’s rules (12). To satisfy linear assumptions, highly skewed variables (absolute skewness >2) were log-transformed based on the Akaike information criterion. Multicollinearity was rigorously screened using a Spearman correlation matrix (threshold |r| > 0.8) and variance inflation factors (<5); for highly collinear homologous indicators (e.g., INR and PT), independent parallel analyses were executed in multivariable models to prevent distortion.

#### Biomarker selection and independent predictive performance

To eliminate redundant information within coaxial indicators, we introduced a stability selection algorithm based on 500 bootstrap resamples with replacement: the indicator with the highest mean area under the curve (AUC) and the lowest standard deviation in the training sets within each group was selected as the representative index. Subsequently, multivariable logistic regression (adjusting for the 11 core covariates) was used to assess independent predictive performance, with the continuous net reclassification improvement (NRI) and integrated discrimination improvement (IDI) calculated via 1,000 bootstrap resamples (13). Adjusted AUCs across indices were compared using the DeLong test, with the Benjamini-Hochberg procedure applied to control the false discovery rate (FDR < 0.05). Dose–response relationships were evaluated using RCS. To ensure model convergence for highly skewed variables, an adaptive knot reduction algorithm (adaptively optimising 3–5 knots based on data distribution) was introduced, dynamically anchoring the reference value (odds ratio = 1) to the median or 25th percentile, with simultaneous testing for overall effects and non-linear trends.

#### Synergistic interaction analysis of inflammation and nutrition

Patients were cross-stratified using optimal cutoffs derived from the Youden index to assess the risk of combined exposure to systemic inflammation and relatively low albumin concentrations. Crucially, the data-derived albumin threshold (43.69 g/L) was applied exclusively to elucidate relative risk gradients for adverse outcomes within this specific cohort; it does not constitute a clinical diagnostic criterion for hypoalbuminaemia or malnutrition. Multiplicative interaction was evaluated via the Wald test of the product term. Given the high incidence of poor outcomes in our cohort (>20%), which could lead to a systematic overestimation of relative risk by odds ratios, additive interaction analysis employed modified Poisson regression with robust standard errors to derive relative risks (RRs). The relative excess risk due to interaction (RERI), attributable proportion (AP), and synergy index (S) were then calculated, with 95% CIs obtained from 2000 bootstrap resamples.

#### Joint model construction and full-pipeline internal validation

A dual-track events-per-variable assessment was performed to ensure modelling feasibility for both binary and ordinal outcomes. Dimensionality reduction was achieved using ordinal least absolute shrinkage and selection operator (LASSO) regression; the penalty parameter (λ) was adaptively optimised through 10-fold cross-validation based on the Bayesian information criterion (BIC). Provided the proportional odds assumption held (Brant test *p* > 0.05), a proportional odds logistic regression model was fitted to generate continuous marginal effect plots for each ordinal outcome level, alongside the construction of a clinical nomogram and decision curve analysis for binary outcomes. To rigorously quantify and correct for optimism bias, 1,000 “full-pipeline” bootstrap internal validation loops were executed. To completely prevent data leakage, LASSO feature selection (including BIC-based hyperparameter tuning) was mandatorily nested and executed independently within each bootstrap loop. We report the optimism-adjusted ordinal C-statistic (using Somers’ D rank correlation), Brier Skill Score, and calibration slope for binary classification.

#### Sensitivity analysis for unmeasured NIHSS confounding

Given the retrospective design of this study and the unavailability of essential baseline covariates—most notably baseline National Institutes of Health Stroke Scale (NIHSS) scores—a three-tiered sensitivity assessment was conducted to establish the robustness of our observational findings: (1) computation of *E*-values to quantify the minimum strength of unmeasured confounding required to entirely nullify the observed associations. In view of the high incidence of unfavourable outcomes in this cohort (22.4%), adjusted odds ratios were converted to approximate relative risks using the transformation algorithm proposed by Ding and VanderWeele prior to *E*-value calculation to avoid overestimating relative risks; for effect estimates with 95% CIs spanning the null value (1.00), the *E*-value for the confidence limit was rigorously defined as 1.00; (2) parametric simulation based on VanderWeele bias formulas to model theoretical effect attenuation following adjustment for residual confounding; and (3) construction of a physiologically restricted subgroup (restricted to systolic blood pressure 140–180 mm Hg, fasting blood glucose 6–10 mmol/L, and age 65–75 years) to evaluate the consistency of effect sizes for key biomarkers within a demographically and clinically homogeneous subset. Crucially, this investigation represents an exploratory association study; therefore, these statistical inferences reflect correlative relationships and do not confer causal attribution.

All statistical analyses were performed using R software (version 4.5.0). Core packages included mice, rms, ordinalNet, pROC, boot, and Hmisc. A two-sided *p* –value < 0.05 was considered statistically significant.

## Results

### Baseline characteristics and data preprocessing

We included 495 patients with clinically and radiologically confirmed AIS, comprising 292 (59.0%) men and 203 (41.0%) women, with a median age of 71 years (IQR 66–80). At the 1-year follow-up, 111 (22.4%) patients had experienced a poor functional outcome (mRS > 2). No deaths occurred during the follow-up period. Univariate analysis revealed that, compared with those having favourable outcomes, patients in the poor outcome group were older (median 77 years vs. 70 years, *p* < 0.001) and had higher prevalences of atrial fibrillation and a smoking history (both *p* < 0.05). Baseline biochemical analyses indicated that the poor outcome group had significantly higher levels of leukocytes, neutrophils, fasting blood glucose, and D-dimer, alongside significantly lower levels of lymphocytes and albumin (all *p* < 0.05). Regarding derived biomarkers, pure inflammatory indices (NLR and SII) and composite inflammatory-nutritional indices (CAR and FAR) were significantly elevated in the poor outcome group (all *p* ≤ 0.002), whereas the PNI was significantly lower (median 48.5 vs. 49.6, *p* = 0.006; Table 1).

**Table 1:** Baseline characteristics (overall and grouped by 1-year mRS).

Variable	mRS ≤ 2	mRS > 2	*p*-value
	*N* = 384	*N* = 111	
Gender, *n* (%)			0.231
Female	152 (40%)	51 (46%)	
Male	232 (60%)	60 (54%)	
Age, *M* (Q1, Q3)	70 (66, 78)	77 (69, 84)	<0.001
Systolic blood pressure (mmHg, *x* ± *s*)	146 ± 20	149 ± 22	0.111
Height, [m, *M* (Q1, Q3)]	1.67 (1.60, 1.72)	1.66 (1.60, 1.72)	0.920
Body weight, [kg, *M* (Q1, Q3)]	68 (60, 75)	65 (60, 75)	0.264
Smoking, *n* (%)			0.045
No	233 (61%)	79 (71%)	
Yes	151 (39%)	32 (29%)	
Hypertension, *n* (%)			0.405
No	74 (19%)	17 (15%)	
Yes	310 (81%)	94 (85%)	
Diabetes mellitus, *n* (%)			0.196
No	201 (52%)	50 (45%)	
Yes	183 (48%)	61 (55%)	
Hyperlipidemia, *n* (%)			0.384
No	155 (40%)	50 (45%)	
Yes	229 (60%)	61 (55%)	
Coronary heart disease, *n* (%)			0.516
No	221 (58%)	60 (54%)	
Yes	163 (42%)	51 (46%)	
Atrial fibrillation, *n* (%)			0.045
No	359 (93%)	97 (87%)	
Yes	25 (6.5%)	14 (13%)	
Internal carotid artery stenosis, *n* (%)			>0.999
No	255 (66%)	74 (67%)	
Yes	129 (34%)	37 (33%)	
Leucocyte, [×10^9^/L, *M* (Q1, Q3)]	6.34 (5.31, 7.73)	7.47 (5.63, 8.60)	0.005
Hemoglobin, [g/L, *M* (Q1, Q3)]	135 (124, 144)	136 (122, 144)	0.608
Blood platelet, [×10^9^/L, *M* (Q1, Q3)]	210 (173, 255)	207 (181, 249)	0.877
Neutrophil, [×10^9^/L, *M* (Q1, Q3)]	4.14 (3.17, 5.21)	4.79 (3.72, 6.30)	<0.001
Lymphocyte, [×10^9^/L, *M* (Q1, Q3)]	1.56 (1.23, 1.99)	1.37 (1.03, 1.87)	0.017
Monocyte, [×10^9^/L, *M* (Q1, Q3)]	0.42 (0.34, 0.53)	0.45 (0.34, 0.57)	0.149
HsCRP (G1), [mg/L, *M* (Q1, Q3)]	1.28 (0.50, 4.65)	3.31 (0.96, 11.00)	<0.001
PT, [sec, *M* (Q1, Q3)]	11.30 (10.80, 11.90)	11.50 (11.00, 12.20)	0.044
APTT, [sec, *M* (Q1, Q3)]	31.0 (29.2, 33.1)	30.2 (28.3, 32.3)	0.023
Fibrinogen, [g/L, *M* (Q1, Q3)]	3.19 (2.80, 3.67)	3.46 (2.94, 4.04)	0.003
D Dimer, [ng/mL, *M* (Q1, Q3)]	144 (88, 237)	244 (144, 474)	<0.001
Potassium, [mmol/L, *M* (Q1, Q3)]	4.00 (3.70, 4.20)	3.95 (3.70, 4.16)	0.290
Sodium, [mmol/L, *M* (Q1, Q3)]	140.0 (138.0, 142.0)	139.6 (137.6, 141.0)	0.109
AST, [U/L, *M* (Q1, Q3)]	17 (13, 24)	19 (13, 31)	0.095
ALT, [U/L, *M* (Q1, Q3)]	18 (14, 23)	20 (15, 30)	0.043
Fasting glucose, [mmol/L, *M* (Q1, Q3)]	6.3 (5.3, 8.3)	6.7 (5.8, 10.1)	0.004
Creatinine, [μmol/L, *M* (Q1, Q3)]	69 (57, 85)	64 (52, 82)	0.045
Uric acid, [μmol/L, *M* (Q1, Q3)]	311 (256, 363)	294 (234, 377)	0.185
Albumin (G3), [g/L, *M* (Q1, Q3)]	41.8 (39.0, 44.3)	40.9 (38.0, 43.6)	0.030
Total cholesterol, [mmol/L, *M* (Q1, Q3)]	4.31 (3.47, 5.29)	4.16 (3.35, 5.09)	0.354
Triglyceride, [mmol/L, *M* (Q1, Q3)]	1.32 (0.99, 1.80)	1.21 (0.95, 1.76)	0.331
HDL, [mmol/L, *M* (Q1, Q3)]	1.08 (0.92, 1.24)	1.03 (0.89, 1.26)	0.370
LDL, [mmol/L, *M* (Q1, Q3)]	2.37 (1.70, 3.11)	2.37 (1.73, 2.94)	0.704
Glycosylated hemoglobin (G3), [%, *M* (Q1, Q3)]	6.25 (5.80, 7.60)	6.55 (5.90, 7.90)	0.277
Homocysteine, [μmol/L, *M* (Q1, Q3)]	13 (11, 16)	14 (11, 18)	0.187
NLR (G1), *M* (Q1, Q3)	2.58 (1.86, 3.52)	3.23 (2.27, 5.05)	<0.001
SII (G1), *M* (Q1, Q3)	531 (366, 790)	654 (470, 1,117)	<0.001
CAR (G2), *M* (Q1, Q3)	0.03 (0.01, 0.11)	0.08 (0.02, 0.25)	<0.001
FAR (G2), *M* (Q1, Q3)	0.076 (0.067, 0.088)	0.082 (0.070, 0.101)	0.002
CHG (G3), *M* (Q1, Q3)	2.56 (2.25, 2.90)	2.66 (2.32, 3.12)	0.070
PNI (G4), *M* (Q1, Q3)	49.6 (46.2, 53.1)	48.5 (44.7, 51.3)	0.006

Death (mRS = 6) accounted for 0 cases, representing 0.0% of the mRS > 2 group. Missing data details are provided in Supplementary Table S1. HCY is provided for descriptive reference only. Natural log-transformed statistics (Mean±SD) for highly skewed indices: ln(NLR): Overall 1.04 ± 0.58, mRS ≤ 2 0.98 ± 0.54, mRS > 2 1.26 ± 0.64; ln(SII): Overall 6.38 ± 0.65, mRS ≤ 2 6.31 ± 0.62, mRS > 2 6.60 ± 0.71; ln(hsCRP): Overall 0.77 ± 1.46, mRS ≤ 2 0.61 ± 1.41, mRS > 2 1.25 ± 1.52; ln(CAR): Overall −2.95 ± 1.49, mRS ≤ 2–3.12 ± 1.44, mRS > 2–2.43 ± 1.56; ln(FAR): Overall −2.54 ± 0.27, mRS ≤ 2–2.56 ± 0.25, mRS > 2–2.46 ± 0.31.

Prior to statistical modelling, extreme values and logical anomalies were excluded based on physiological plausibility checks (Supplementary Table S1). Little’s MCAR test indicated that data were not missing completely at random (*p* < 0.001); consequently, variables with missing data rates between 5 and 30% underwent multiple imputation under the missing at random assumption (Supplementary Table S2). Right-skewed indicators, such as NLR and SII, were log-transformed based on the Akaike information criterion (Supplementary Table S3). To address highly collinear coagulation metrics (e.g., prothrombin time and international normalised ratio, *r* = 0.945), isolated analyses were executed in subsequent modelling procedures to prevent multicollinearity (Supplementary Tables S4, S5).

### Independent prognostic value and representative biomarker selection

Univariate logistic regression analysis demonstrated that all nine evaluated biochemical derived indices were significantly associated with poor functional outcomes at 1 year (all *p* < 0.05; Supplementary Table S6), among which hsCRP exhibited the highest univariate discriminative performance (AUC = 0.652), while ALB and PNI emerged as protective factors (Figure 2A). Following adjustment for the 11 clinical covariates (baseline model AUC = 0.752), we utilised the continuous net reclassification improvement (NRI) and integrated discrimination improvement (IDI), alongside 500 bootstrap resamples, to select representative features (Table 2).

**Figure 2:**
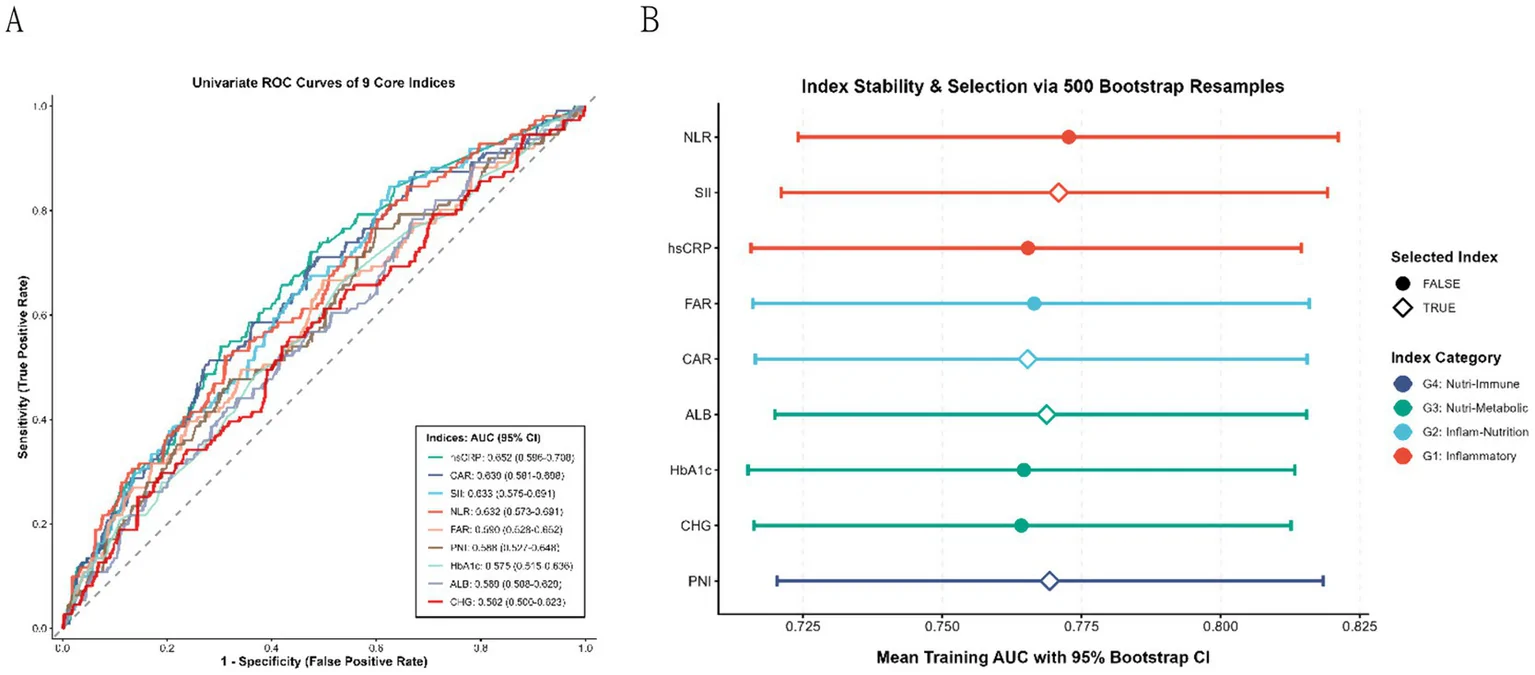
Identification and robust selection of prognostic biomarkers. **(A)** Univariate Receiver Operating Characteristic (ROC) curves evaluating the discriminatory ability of the nine derived inflammatory, nutritional, and metabolic indices for predicting 1-year poor functional outcomes (mRS > 2). **(B)** Forest plot illustrating the predictive stability and selection of representative indices within each pathophysiological axis, rigorously validated through 500 bootstrap resampling iterations. Points represent the mean training AUC, and error bars indicate the 95% bootstrap confidence intervals. Selected top-performing indices are highlighted with diamond markers.

**Table 2:** Multivariable incremental value and bootstrap-based selection of core indices.

Category/Index	Adjusted OR (95% CI)	Adjusted AUC	NRI total (95% CI)	IDI (95% CI)	Bootstrap train AUC (95% CI)	Selected?
G1: Inflammatory						
NLR	1.42 (1.14–1.76)**	0.758	0.331 (0.056–0.592)	0.023 (0.004–0.058)	0.773 (0.724–0.821)	Yes*
SII	1.33 (1.07–1.65)*	0.757	0.403 (0.133–0.623)	0.015 (0.001–0.050)	0.771 (0.721–0.819)	No
hsCRP	1.13 (0.92–1.40)	0.752	0.031 (−0.177–0.335)	0.003 (0.000–0.030)	0.765 (0.716–0.814)	No
G2: Inflam-nutrition						
CAR	1.14 (0.91–1.44)	0.752	0.044 (−0.174–0.329)	0.003 (0.000–0.024)	0.765 (0.716–0.816)	Yes
FAR	1.27 (1.00–1.62)	0.751	0.146 (−0.119–0.426)	0.011 (0.000–0.036)	0.766 (0.716–0.816)	No
G3: Nutri-metabolic						
ALB	0.77 (0.59–1.01)	0.757	0.133 (−0.058–0.383)	0.008 (0.000–0.027)	0.769 (0.720–0.815)	Yes
HbA1c	1.21 (0.86–1.72)	0.752	0.101 (−0.106–0.373)	0.003 (0.000–0.020)	0.765 (0.715–0.813)	No
CHG	0.97 (0.66–1.41)	0.752	−0.022 (−0.119–0.281)	0.000 (0.000–0.011)	0.764 (0.716–0.813)	No
G4: Nutri-immune						
PNI	0.75 (0.58–0.98)*	0.757	0.203 (−0.009–0.491)	0.010 (0.000–0.036)	0.769 (0.720–0.818)	Yes

OR represents the odds ratio per 1 Standard Deviation (SD) increment of each index. Multivariable models were adjusted for 11 core clinical covariates (Age, Gender, BMI, Hypertension, Diabetes, Smoking, SBP, Fasting Glucose, Creatinine, Hemoglobin, and ln_D-Dimer). The baseline model comprising these 11 covariates yielded an AUC of 0.752. Continuous Net Reclassification Improvement (NRI) and Integrated Discrimination Improvement (IDI) metrics, along with their 95% CIs, were derived from 1,000 bootstrap iterations. Model stability (Bootstrap Train AUC) was evaluated via 500 resamples. *p* < 0.05, **p < 0.01 for the adjusted OR. * Clinical Override: Although SII.

Internal validation revealed that the mean AUC for all candidate indicators across the resampled datasets ranged narrowly from 0.764 to 0.773 (Figure 2B). Based on their incremental multivariable value, CAR, ALB, and PNI were identified as the representative indices for dimensions G2, G3, and G4, respectively. Within the pure inflammatory dimension (G1), the NLR yielded a more robust independent effect estimate (adjusted odds ratio [OR] 1.42, 95% CI 1.14–1.76; *p* < 0.01) and superior IDI improvement (0.023); consequently, it was confirmed as the representative feature for the G1 dimension (Table 2). Pairwise DeLong tests indicated no statistically significant differences in the overall discriminative performance among these nine independent biomarkers (all *p* > 0.05; Supplementary Table S7).

### Continuous dose–response relationships and non-linear inference

Following adjustment for the 11 clinical covariates, RCS models were constructed to evaluate the continuous dose–response relationships between the biomarkers and poor outcomes (Figure 3; Supplementary Table S8). Using the 25th percentile as the reference, the adjusted odds ratios (ORs) for NLR (overall *p* = 0.008; non-linear *p* = 0.367) and SII (overall *p* = 0.009; non-linear *p* = 0.068) exhibited a monotonic positive trend with increasing continuous levels.

**Figure 3:**
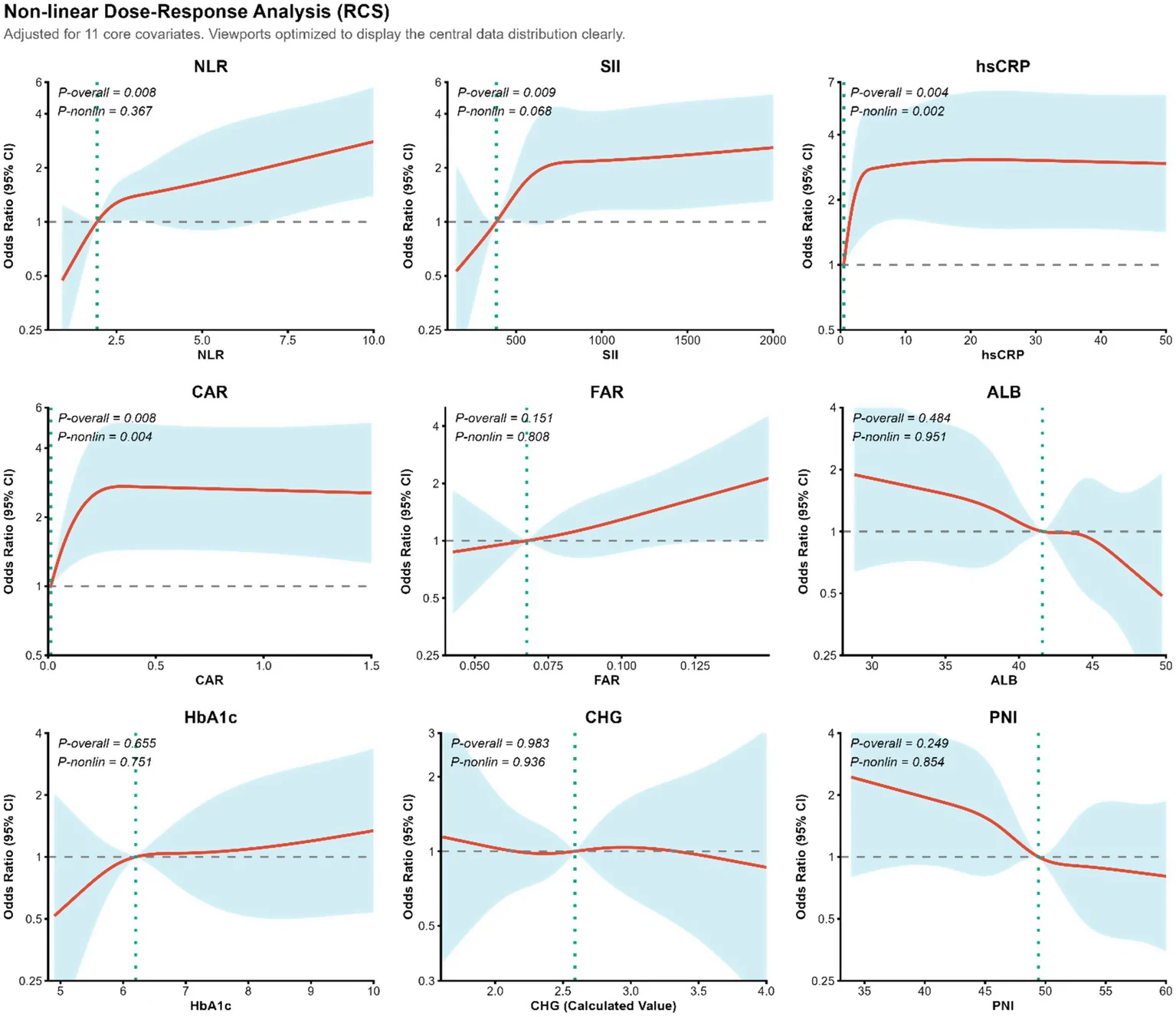
Continuous non-linear dose–response analysis using restricted cubic splines. The plots demonstrate the adjusted odds ratios (ORs, solid red lines) and 95% confidence intervals (shaded blue areas) for the association between nine core derived indices and the risk of poor outcome. Models were comprehensively adjusted for 11 core clinical covariates (including age, gender, stroke severity proxies, and comorbidities). Reference values (OR = 1.0, vertical dotted lines) were dynamically anchored at the 25th or 50th percentiles based on data skewness. Adaptive knot placement was utilised to ensure model convergence for highly skewed variables.

Furthermore, hsCRP and CAR demonstrated significant non-linear associations (non-linear *p* = 0.002 and *p* = 0.004, respectively). RCS curves revealed that the risk trajectory for hsCRP exhibited a plateau effect: within the 0.5–10 mg/L range, the risk of a poor outcome increased significantly (peak OR \approx1.62), whereas the OR plateaued beyond this threshold. The dose–response curve for CAR displayed an inverted U-shape, reaching a peak risk at a ratio of approximately 0.5 (OR \approx1.44) before subsequently levelling off (Figure 3). The overall continuous main effects for FAR and pure nutritional-metabolic indicators (e.g., ALB and HbA1c) did not reach statistical significance (overall *p* > 0.05 for all).

### Cross-classification and synergistic interaction analysis

Exploratory cross-stratification of patients was done using data-derived Youden index cutoffs (NLR 3.22; albumin 43.69), and adjusted relative risks (RRs) were estimated via a modified Poisson regression model with robust standard errors (Figure 4; Table 3). Compared with the low-inflammation and high-nutrition reference group (G1 Low/G3 High), neither isolated high inflammation nor an isolated low albumin status significantly increased the risk of an unfavourable prognosis. However, the concomitant presence of a high NLR and low albumin concentration was associated with a significantly elevated risk of unfavourable outcomes (adjusted RR 2.45 [95% CI 1.33–4.49]). On the absolute risk scale, the incidence of unfavourable outcomes escalated from 11.5% in the reference group to 36.9% in the combined exposure group (absolute risk difference 25.4% [95% CI 13.2–37.6]).

**Figure 4:**
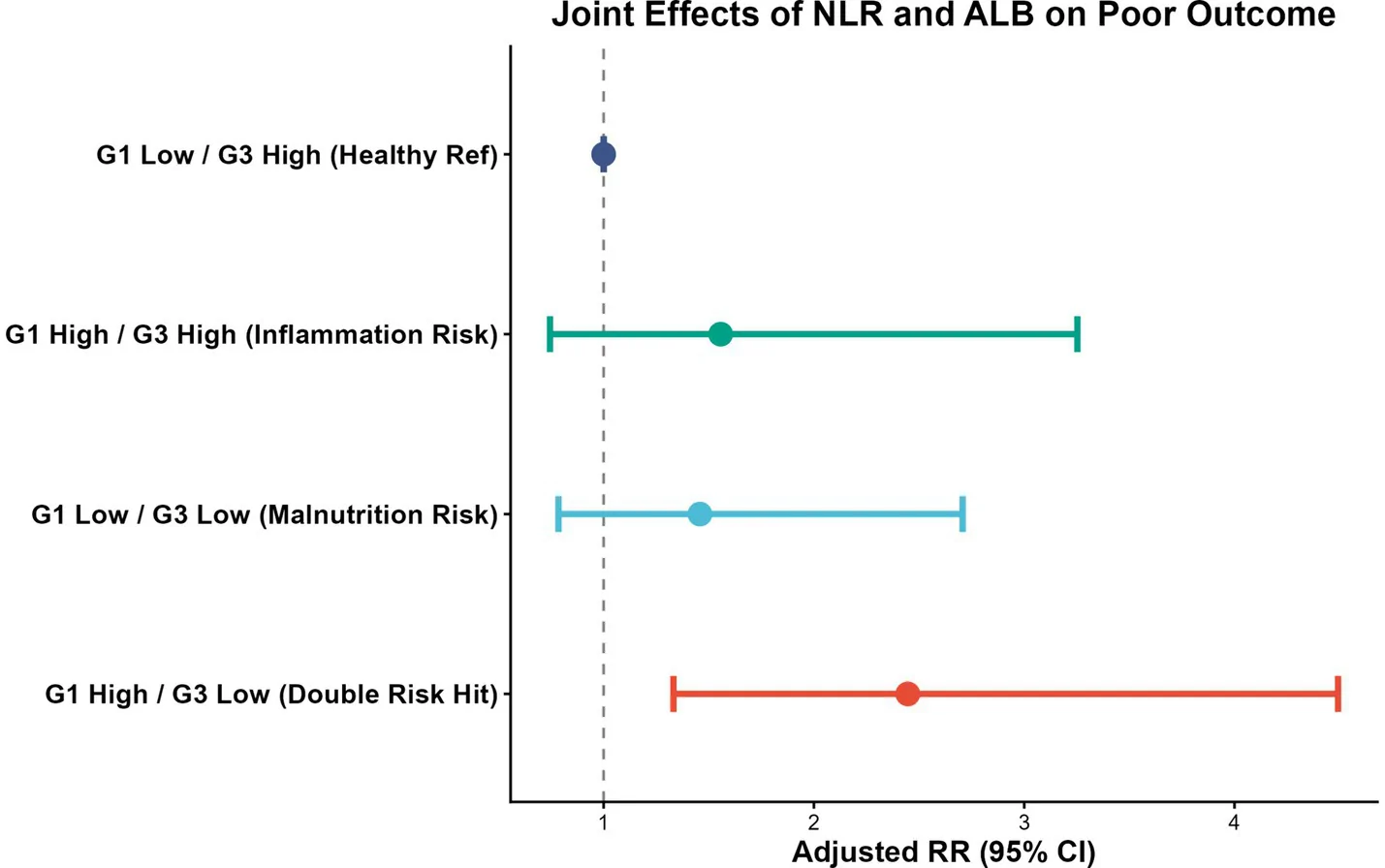
Joint high-risk association between systemic inflammation and nutritional status. Forest plot demonstrating the joint effects of the Neutrophil-to-Lymphocyte Ratio (NLR) and Albumin (ALB) on the risk of poor outcome. Patients were cross-classified based on data-derived exploratory Youden index cutoffs. The analysis highlights a significant joint high-risk association when severe systemic inflammation is coupled with poor nutritional status, adjusted for core clinical covariates. Note: Neither multiplicative (*p* = 0.856) nor additive interaction (RERI 95% CI crosses 0) reached statistical significance, suggesting a hypothesis-generating trend rather than confirmed synergy.

**Table 3:** Comprehensive interaction analysis.

Groups/interaction metrics	Total (*N*)	mRS > 2, *n* (%)	Adjusted RR (95% CI)
Subgroup analysis (cross-classification)			
G1 low/G3 high (healthy ref)	96	11 (11.5%)	Ref (1.00)
G1 high/G3 high (inflammation risk)	55	13 (23.6%)	1.56 (0.74–3.25)
G1 low/G3 low (malnutrition risk)	222	42 (18.9%)	1.46 (0.79–2.71)
G1 high/G3 low (double risk hit)	122	45 (36.9%)	2.45 (1.33–4.49)
Multiplicative interaction			
Product term (NLR × ALB)			1.08 (0.48–2.42) [*p* = 0.856]
Additive interaction			
Relative excess risk due to interaction (RERI)			0.432 (−1.193 to 1.519)
Attributable proportion (AP)			0.177 (−0.383 to 0.593)
Synergy index (S)			1.426 (−9.271 to 10.318)
Additive conclusion			No Significant Additive Interaction

Cutoffs (Youden): NLR = 3.22, ALB = 43.69. Power (OR = 2.0) = 36.2%. Models adjusted for 11 core covariates. mRS > 2 incidence ≥20%; Modified Poisson with robust standard errors used for direct Adjusted RR. Additive interaction 95% CIs were estimated via 2,000 bootstrap resamples.

displayed a marginally smaller standard deviation during bootstrap resampling, NLR was selected as the representative for the inflammatory group (G1) due to its higher mean absolute AUC (0.773 vs. 0.771), superior IDI improvement (0.023 vs. 0.015), and broader established clinical consensus in neuroinflammation.

Interaction testing revealed no statistically significant interaction on the multiplicative scale (*p* = 0.856). On the additive scale, although point estimates (RERI 0.432; AP 0.177) suggested a positive additive trend, all 95% CIs spanned the null value (e.g., RERI 95% CI –1.193 to 1.519), thereby failing to substantiate a statistically significant synergistic interaction. A post-hoc analysis indicated that the statistical power to detect this interaction within the current cross-stratified subgroups was merely 36.2%; consequently, this combined high-risk association should be interpreted strictly as a hypothesis-generating trend.

### Joint model derivation, internal validation, and decision utility

As presented in Table 4, four representative derived indices (NLR, CAR, albumin, and PNI) and 11 clinical covariates were incorporated into an initial comprehensive model. To assess potential mathematical coupling bias arising from shared algebraic components (e.g., albumin and lymphocytes), rigorous collinearity diagnostics were done. The variance inflation factor (VIF) for PNI reached 5.38 (exceeding the conventional collinearity threshold of 5.0), and the VIF for albumin was 4.88. Belsley collinearity diagnostics provided definitive mathematical evidence of this structural overlap: at the 15th dimension (condition index 6.06), albumin and PNI shared variance decomposition proportions of 76.7 and 81.8%, respectively. This finding confirmed that forcing these structurally homologous indices into a single statistical framework would inevitably introduce substantial information redundancy and model distortion.

**Table 4:** Multivariable association models (standardized units).

Variable	Ordinal model POR (95% CI)	Ordinal *p*-value	Binary model OR (95% CI)	Binary *p*-value
Age	1.38 (1.14–1.67)	<0.001	1.45 (1.12–1.89)	0.006
Gender	0.92 (0.63–1.34)	0.668	1.09 (0.64–1.86)	0.742
BMI	0.80 (0.67–0.95)	0.012	0.70 (0.54–0.92)	0.009
Hypertension	1.40 (0.90–2.16)	0.132	1.32 (0.68–2.55)	0.409
Diabetes	1.04 (0.71–1.52)	0.841	1.21 (0.70–2.10)	0.494
Smoking	0.90 (0.61–1.31)	0.575	0.70 (0.40–1.23)	0.211
SBP	1.04 (0.88–1.23)	0.613	1.09 (0.86–1.38)	0.486
FastGlucose	1.23 (1.01–1.49)	0.043	1.38 (1.08–1.76)	0.011
Creatinine	0.86 (0.72–1.02)	0.077	0.80 (0.60–1.07)	0.131
Hb	1.14 (0.95–1.38)	0.168	1.40 (1.07–1.84)	0.016
ln_DDimer	1.20 (1.00–1.43)	0.049	1.58 (1.19–2.11)	0.002
NLR	1.57 (1.25–1.98)	<0.001	1.52 (1.16–1.99)	0.002
CAR	0.96 (0.78–1.19)	0.729	0.94 (0.72–1.23)	0.639
ALB	0.65 (0.45–0.93)	0.018	0.65 (0.39–1.09)	0.103
PNI	1.35 (0.93–1.98)	0.117	1.23 (0.73–2.08)	0.440

Variables analyzed under standard clinical framework. For continuous variables, Odds Ratios (OR) and Proportional Odds Ratios (POR) represent the risk per 1 Standard Deviation (SD) increment. Binary covariates remain 0/1 coded. Outcomes defined as ordinal mRS (0–5) and binary mRS (>2). Binary model Nagelkerke *R*^2^ = 0.225, Hosmer–Lemeshow *p* = 0.140.

Because conventional logistic regression is not robust against such collinearity, we used a bootstrap-LASSO algorithm with 1,000 resamples for feature dimension reduction. As depicted in Figure 5A, the L1 penalty of the LASSO algorithm successfully identified and shrank this algebraic redundancy: core clinical covariates (e.g., log D-dimer 99.9%; age 99.3%) and the purely cellular index NLR (99.2%) maintained exceptionally high inclusion frequencies across resamples; conversely, owing to its composite nature incorporating both albumin and lymphocytes and its high structural overlap with albumin and NLR, the inclusion frequency of PNI plummeted to 35.5% (well below the prespecified 50% threshold for core feature retention). Consequently, the dimension reduction algorithm automatically eliminated PNI, retaining 14 features for subsequent modelling.

**Figure 5:**
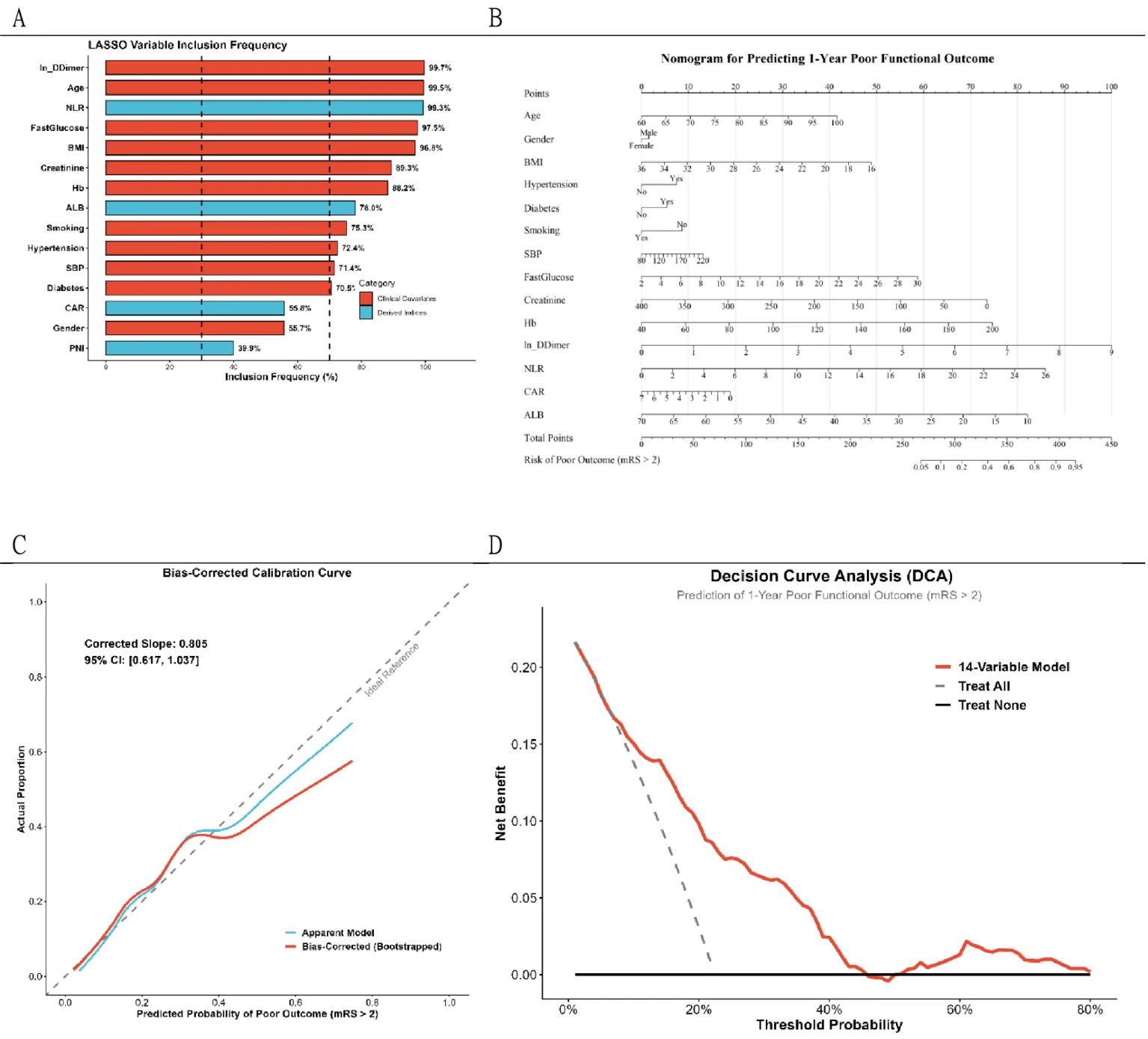
Construction, rigorous internal validation, and clinical utility of the joint prediction model. **(A)** Variable inclusion frequency determined by ordinal LASSO regression across 1,000 bootstrap iterations, proving the feature selection stability. **(B)** A clinical nomogram integrating selected derived indices and clinical covariates to predict the individualised probability of 1-year poor functional outcomes. **(C)** Bootstrap bias-corrected calibration curve showing optimal agreement between the predicted probabilities and actual observations, effectively avoiding risk overestimation. **(D)** Decision Curve Analysis (DCA) demonstrating that the 14-variable joint model provides a superior net clinical benefit across a wide range of threshold probabilities compared to “treat-all” or “treat-none” strategies.

Prior to constructing the final clinical prediction model, statistical feasibility was assessed based on the events per variable (EPV) ratio. Because the EPV for the smallest category in an ordinal multivariable model was only 1.87—posing an exceedingly high risk of overfitting and model non-convergence—clinical risk prediction in this study was strictly restricted to the binary endpoint (mRS > 2; EPV 7.40). A clear distinction must be made here: the initial 15-variable model presented in Table 4 was constructed solely for aetiological association inference and to demonstrate baseline collinearity. By contrast, the final nomogram intended for individualised clinical risk prediction (Figure 5B) incorporated only the 14 non-redundant core predictors retained after LASSO dimension reduction.

To rigorously assess the true clinical performance of this 14-variable predictive model, we implemented a full-pipeline nested internal validation strategy to completely preclude data leakage. Specifically, multiple imputation, hyperparameter tuning, and variable selection were strictly nested within 1,000 bootstrap iterations (Supplementary Table S9). The results yielded an optimism-corrected C-statistic of 0.724 (95% CI 0.700–0.754) and an overall Brier score of 0.1572 (95% CI 0.1488–0.1585), indicating acceptable discrimination and good overall predictive accuracy. Crucially, the bias-corrected calibration intercept was 0.016 (95% CI –0.358 to 0.531) and the calibration slope was 1.004 (95% CI 0.715–1.450). This calibration slope is exceptionally close to the ideal value of 1.0, suggesting that the penalised shrinkage effect of the LASSO algorithm successfully counteracted the systematic risk overestimation inherent to a low EPV, thereby ensuring high agreement between predicted probabilities and actual observations (Figure 5C). Nonetheless, the wide CI of the slope indicates that vigilance regarding residual overfitting risk remains necessary in the context of a small sample size. Decision curve analysis confirmed that, across a threshold probability range of approximately 5 to 45%, this 14-variable dimension-reduced model provided tangible and robust clinical net benefit compared with either the treat-all or treat-none strategies (Figure 5D).

### Sensitivity analyses and robustness to unmeasured confounding

Given the absence of recorded baseline National Institutes of Health Stroke Scale (NIHSS) scores in this cohort, sensitivity analyses were performed to evaluate the potential impact of unmeasured confounding (Table 5; Supplementary Table S11S1). *E*-value analysis indicated that, following the conversion of the odds ratio to an approximate relative risk, an unmeasured confounder would require a minimum association of 1.90 with both the core predictor NLR and the unfavourable outcome to completely nullify the observed main effect. Although the primary model was adjusted for relevant physiological parameters, the theoretical possibility remains that a single residual confounder could invalidate the observed association. This caveat is particularly pertinent given that association strengths for potent unmeasured confounders, such as the omitted baseline NIHSS score, are frequently reported in the literature to range between 1.3 and 2.1. *E*-value analysis indicated that, following the conversion of the odds ratio to an approximate relative risk, an unmeasured confounder would require a minimum association of 1.90 with both the core predictor NLR and the unfavourable outcome to completely nullify the observed main effect. Although the primary model was adjusted for relevant physiological parameters, the theoretical possibility remains that a single residual confounder could invalidate the observed association. This caveat is particularly pertinent given that association strengths for potent unmeasured confounders, such as the omitted baseline NIHSS score, are frequently reported in the literature to range between 1.3 and 2.1.

**Table 5:** Sensitivity analyses for unmeasured confounding (baseline NIHSS) assessing the robustness of inflammatory and nutritional indices.

Biomarker index	Full sample OR (95% CI)	*E*-value (Point/CI limit)	Homogeneous subgroup OR (95% CI)	Effect size relative diff	Synthesized robustness
NLR	1.41 (1.16–1.72)	1.90/1.48	1.76 (1.00–3.31)	25.1%	Highly robust
CAR	1.22 (1.01–1.51)	1.60/1.07	1.38 (0.63–2.82)	13.2%	Moderately robust
ALB	0.81 (0.64–1.02)	1.64/1.00	0.93 (0.39–2.60)	14.4%	Moderately robust
PNI	0.78 (0.62–0.98)	1.73/1.14	0.73 (0.30–1.74)	6.5%	Moderately robust

(1) *E*-value Analysis: Calculated based on Ding and VanderWeele (27). The *E*-value represents the minimum strength of association an unmeasured confounder (e.g., baseline NIHSS) must have with both the index and the outcome to fully explain away the observed association. *E*-value > 2.1 indicates high robustness, exceeding plausible confounding caps reported in stroke literature. (2) Homogeneous Severity Subgroup: Restricted to Age 65–75, SBP 140–180 mmHg, and Fasting Glucose 6.0–10.0 mmol/L (*N* = 58, 11.7%) to artificially minimise baseline severity heterogeneity. Despite being statistically underpowered, all indices maintained consistent effect directions with relative differences ≤ 30%, suggesting the main findings are not entirely driven by unmeasured severity confounding.

Probabilistic simulations revealed that under scenarios assuming strong residual confounding (*R*^2^ = 0.25), the direction of the risk effects for core biomarkers such as NLR (simulated OR 1.25) and CAR was not reversed, with reductions in effect estimates ranging from 11.1 to 12.5% (Supplementary Table S11). Further restriction of the study population to a subgroup defined by stringent physiological parameters (age 65–75 years, systolic blood pressure 140–180 mm Hg, and fasting blood glucose 6.0–10.0 mmol/L; *n* = 58) inherently compromised statistical power. Nevertheless, the direction of the point estimates for all core indices remained consistent, and the relative differences in effect sizes were maintained within 30% (Table 5). These multidimensional sensitivity analyses confirmed the robustness of our observational findings.

## Discussion

Through a systematic methodological re-evaluation, this study elucidates the value of derived haematological indices in assessing the prognosis of AIS. After maximally mitigating mathematical coupling bias via theoretical axis separation and the LASSO dimension reduction algorithm, the purely cellular index—the neutrophil-to-lymphocyte ratio (NLR)—demonstrated a more independent and central predictive utility compared with composite indices (e.g., SII or PNI). Concurrently, non-linear trajectory analysis revealed the physiological limits of inflammatory and nutritional indices during the acute phase, whereas additive interaction analysis preliminarily quantified the joint risk trend associated with a high inflammatory response superimposed on relatively low circulating protein levels. The resulting parsimonious model substantially enhances the stability and pragmatism of point-of-care assessments without compromising net clinical benefit.

A plethora of previous studies have conventionally incorporated derived indices—such as NLR, SII, CAR, and PNI—into the same multivariable models for competitive evaluation. This research paradigm overlooks the inherent sharing of fundamental algebraic components (e.g., lymphocyte count or albumin) among these indices, inevitably introducing mathematical coupling bias, which engenders severe collinear distortion and false-positive associations (11, 14). By rigorously defining pathophysiological axes and employing bootstrap-LASSO for feature dimensionality reduction, the present study effectively decoupled this redundant information. Composite indices like the PNI were eliminated due to their failure to provide incremental information beyond their constituent variables, whereas the NLR was robustly retained (15). This statistical phenomenon is underpinned by robust biological mechanisms: the disruption of the blood–brain barrier during the acute phase of AIS is primarily driven by matrix metalloproteinases released by neutrophils, whereas the rapid apoptosis of peripheral lymphocytes is a hallmark of stroke-induced immunodepression syndrome (SIDS) (16). Compared to the PNI, which is confounded by the lagging effect of albumin’s long half-life, the purely cytological NLR is more acutely sensitive to the initiating events of acute-phase immunological imbalance.

Beyond circumventing structural overlap, discarding conventional linear assumptions is equally pivotal to uncovering true biological phenomena. RCS analysis revealed a marked plateau effect in the association between hsCRP and poor outcomes, whereas CAR exhibited an inverted U-shaped trajectory. Such non-linear distributions profoundly reflect the physiological limits of the body’s acute stress response. When systemic inflammation breaches a specific threshold, the hepatic capacity to synthesise acute-phase proteins approaches saturation, precipitating a flattening of the risk curve (17). A plausible pathophysiological explanation for the risk attenuation at extremely high CAR values is that patients at this extreme have largely descended into end-stage cachexia or severe hepatic synthetic failure; the body loses both the substrate and the capacity to continuously upregulate inflammatory proteins, entering a state of so-called immune paralysis (18, 19). Forcibly fitting a linear model to this frailty-driven heterogeneity would obscure the true risk trajectories of high-risk populations, consequently leading to the misalignment of future clinical intervention cut-offs (20).

Quantitative exploration of the “double-hit” hypothesis elucidated a pronounced risk profile associated with concurrent exposure to elevated systemic inflammation and low albumin concentrations (relative risk [RR] 2.45; absolute risk increase 25.4%). However, a strict delineation must be drawn between an epidemiological joint association and statistically confirmed synergism. Constrained by limited statistical power within the cross-classified subgroups (36.2%), neither multiplicative (*p* = 0.856) nor additive interaction—the latter holding greater clinical relevance for guiding combinatorial interventions (95% CI for the relative excess risk due to interaction [RERI] encompassing zero)—attained statistical significance. The synergy index (S > 1) merely indicates a potential positive additive trend. Consequently, this coupled inflammatory–nutritional effect should presently be interpreted strictly as hypothesis-generating. Given the retrospective study design, the absence of prospective nutritional assessment scales, and the reliance on data-derived thresholds, the present findings are insufficient to substantiate the routine clinical implementation of exogenous intensive nutritional interventions. Future interventional strategies targeting the modulation of excessive acute-phase inflammation in conjunction with metabolic homeostasis support urgently necessitate definitive validation in adequately powered, large-scale, prospective trials utilising established clinical cut-offs (21, 22).

In terms of clinical translation, the parsimonious prognostic model—comprising solely age, log-transformed D-dimer, and NLR—exhibited exceptional generalisability. Decision curve analysis (DCA) substantiated the net clinical benefit of this model across a broad spectrum of threshold probabilities (5). It must be emphasised that this model is not intended to supplant standard neurological assessments (e.g., the NIHSS), but rather to serve as a potent adjunct. In settings lacking specialist examination capabilities (such as pre-hospital emergency care or primary care triage), or when patients are uncooperative due to severe aphasia or impaired consciousness, this model provides an alternative risk stratification pathway reliant on objective, low-cost haematological parameters.

Several limitations of this study warrant consideration. First, the retrospective design inherently precludes the elimination of unmeasured confounding. Crucial covariates, including baseline National Institutes of Health Stroke Scale (NIHSS) scores, infarct volume, and reperfusion history, were unavailable in this cohort. Although proxy variables capable of partially reflecting disease severity—such as age, blood pressure, and glycaemia—were incorporated into the models to constrain the potential for systematic bias (23), these indices cannot entirely substitute for direct neurological deficit assessments. To quantify the impact of this potential confounding, we conducted a sensitivity analysis: adjusting for the incidence rate (22.4%), the *E*-value for the NLR was calculated at 1.90. According to VanderWeele’s causal inference framework (24), an unmeasured confounder such as the NIHSS would need to be associated with both the exposure (NLR) and the outcome by a relative risk exceeding 1.90 to entirely nullify the observed independent effect. Given that baseline neurological function is a robust determinant of stroke prognosis, its effect size could easily surpass this threshold. Consequently, residual confounding cannot be ruled out; our findings must be strictly interpreted as an exploratory association rather than establishing causality.

Second, the construct validity of acute-phase serum albumin as an exposure index is limited. The acute phase of ischaemic stroke is frequently accompanied by capillary leakage induced by systemic inflammation and endothelial injury, during which albumin acts predominantly as a negative acute-phase reactant. Fluctuations in albumin concentrations are profoundly influenced by pre-stroke hydration status, occult infections, and hepatic and renal metabolism, and thus cannot be simplistically equated with baseline nutritional reserves. Furthermore, the data-derived cut-off value in our study (43.69 g/L) falls within the conventional physiological range. In the absence of standardized nutritional assessment tools, such as the GLIM criteria or NRS-2002, and without adjusting for the haemodilution effects of intravenous fluid resuscitation, this threshold cannot serve as a clinical diagnostic criterion for malnutrition. Therefore, alterations in serum albumin concentrations in this context should be more appropriately interpreted as a marker of acute-phase protein metabolic imbalance, rather than a direct indication for intensive nutritional intervention.

Finally, the single-centre data structure restricts the spatial transportability of our models; these findings require external validation in multicentre cohorts (25). Simultaneously, single-timepoint cross-sectional data preclude the capture of the dynamic evolution of these biomarkers. Future research should consider incorporating latent class trajectory modelling to elucidate the time-dependent characteristics of these pathological networks (26). Methodologically, although least absolute shrinkage and selection operator (LASSO) regression was utilised to mitigate algebraic collinearity among variables, isolating latent correlations completely within conventional multivariable models remains challenging. Subsequent studies could attempt to integrate latent variable models to directly extract core biological components, thereby affording a more precise assessment of their prognostic value.

In conclusion, circumventing mathematical coupling bias and the pitfalls of linear presumptions is a fundamental prerequisite for the precise evaluation of peripheral biomarkers in stroke. The independent prognostic value of the pure inflammatory index, NLR, supersedes that of composite indices constrained by structural overlap. A parsimonious prognostic model constructed upon core signals provides an objective and cost-effective methodological rationale for early risk stratification in acute ischaemic stroke. Future comprehensive interventional pathways targeting the synergistic maintenance of anti-inflammatory responses and metabolic homeostasis during the acute phase of stroke warrant prudent validation in prospective cohorts and clinical trials incorporating multidimensional nutritional assessments.

## Data Availability

The datasets used and/or analyzed during the current study are available from the corresponding author upon reasonable request.
