# Supplementary material for: Interactive effects of systemic inflammation and nutritional status on 1-year functional outcomes in acute ischaemic stroke: a multidimensional biomarker cohort study
Source: Front Nutr. 2026 Oct 2;13:1969226. doi: 10.3389/fnut.2026.1969226 (PMC13671964; doi:10.3389/fnut.2026.1969226)
Supplement: Supplementary file 1 [file Table_1.DOCX]

**Table S1. Physiological Plausibility Assessment of Baseline Variables**

| **Variable** | **N_Valid** | **Zero Lymphocyte (n)** | **Below_Limit** | **Above_Limit** | **Action** |
| --- | --- | --- | --- | --- | --- |
| NLR | 493 | 0 | 0 | 0 | Passed |
| SII | 493 | 0 | 0 | 1 | ⚠ Check Raw Data |
| hsCRP | 381 | 0 | 0 | 0 | Passed |
| Albumin | 492 | 0 | 1 | 3 | ⚠ Check Raw Data |
| Fibrinogen | 485 | 0 | 1 | 1 | ⚠ Check Raw Data |
| TC | 486 | 0 | 0 | 1 | ⚠ Check Raw Data |
| HDL | 486 | 0 | 1 | 0 | ⚠ Check Raw Data |
| FastGlucose | 490 | 0 | 0 | 0 | Passed |
| CHG | 482 | 0 | 0 | 11 | ⚠ Check Raw Data |
| PNI | 490 | 0 | 1 | 1 | ⚠ Check Raw Data |
| BMI | 405 | 0 | 0 | 0 | Passed |
| Height | 430 | 0 | 0 | 0 | Passed |
| Weight | 406 | 0 | 0 | 0 | Passed |
| PT | 485 | 0 | 0 | 0 | Passed |
| APTT | 485 | 0 | 0 | 0 | Passed |
| INR | 485 | 0 | 0 | 1 | ⚠ Check Raw Data |

*Note: Baseline Death Cases (mRS=6): n=0, representing 0.0% of the adverse outcome group (mRS > 2).*

**Table S2. Missing Data Diagnostics and Imputation Strategy**

| **Variable** | **Missing Percentage** | **Imputation Strategy** |
| --- | --- | --- |
| HbA1c | 23.63% | MICE |
| CAR | 23.43% | MICE |
| hsCRP | 23.03% | MICE |
| BMI | 18.18% | MICE |
| Homocysteine | 7.47% | MICE |
| ln_DDimer | 4.04% | CCA |
| FAR | 2.62% | CCA |
| CHG | 2.62% | CCA |
| PT | 2.02% | CCA |
| APTT | 2.02% | CCA |
| Fibrinogen | 2.02% | CCA |
| TC | 1.81% | CCA |
| HDL | 1.81% | CCA |
| FastGlucose | 1.01% | CCA |
| PNI | 1.01% | CCA |
| HALP | 1.01% | CCA |

*Note: Missing data mechanisms were evaluated using Little's MCAR Test (Chi-square=736.95, p<0.001), indicating data were Missing at Random (MAR). Based on the missingness threshold, variables with <5% missingness were handled using Complete Case Analysis (CCA), whereas variables with 5%-30% missingness underwent Multiple Imputation by Chained Equations (MICE) using Predictive Mean Matching (m=20).*

**Table S3. Distributional Assessment and Transformation Strategy**

| **Variable** | **Shapiro_P** | **Skewness** | **AIC_Original** | **AIC_Log** | **Final_Decision** | **Model_Note** |
| --- | --- | --- | --- | --- | --- | --- |
| NLR | <0.001 | 3.531 | 512.3 | 507.8 | Log Transformed (AIC Win) | RCS Ref: 25th Pct |
| SII | <0.001 | 6.733 | 518.6 | 510.5 | Log Transformed (AIC Win) | RCS Ref: 25th Pct |
| hsCRP | <0.001 | 5.797 | 426.1 | 416.2 | Log Transformed (AIC Win) | RCS Ref: 25th Pct |
| CAR | <0.001 | 6.219 | 424.7 | 414.3 | Log Transformed (AIC Win) | RCS Ref: 25th Pct |
| FAR | <0.001 | 3.308 | 503.5 | 503.3 | Log Transformed (AIC Win) | RCS Ref: 25th Pct |
| ALB | <0.001 | 0.045 | - | - | Original | Mean/Median |
| HbA1c | <0.001 | 1.336 | - | - | Original (Log in Sensitivity) | Mean/Median |
| CHG | <0.001 | 0.763 | - | - | Pre-specified: Raw | Mean/Median |
| PNI | <0.001 | 0.077 | - | - | Original | Mean/Median |
| PLR | <0.001 | 3.258 | 522.0 | 521.5 | Log Transformed (AIC Win) | RCS Ref: 25th Pct |
| MLR | <0.001 | 14.431 | 526.4 | 519.7 | Log Transformed (AIC Win) | RCS Ref: 25th Pct |
| SIRI | <0.001 | 6.957 | 520.0 | 510.6 | Log Transformed (AIC Win) | RCS Ref: 25th Pct |
| HCY | <0.001 | 3.669 | 484.7 | 483.8 | Log Transformed (AIC Win) | RCS Ref: 25th Pct |
| Age | <0.001 | 0.537 | - | - | Original | Mean/Median |
| BMI | 0.007 | 0.348 | - | - | Original | Mean/Median |
| SBP | 0.131 | 0.188 | - | - | Original | Mean/Median |
| FastGlucose | <0.001 | 2.089 | 517.7 | 517.2 | Log Transformed (AIC Win) | RCS Ref: 25th Pct |
| ln_DDimer | <0.001 | -0.470 | - | - | Pre-specified: ln(x+1) | Mean/Median |
| Creatinine | <0.001 | 3.630 | 527.7 | 525.0 | Log Transformed (AIC Win) | RCS Ref: 25th Pct |

*Note: Absolute Skewness > 2 triggered AIC comparison between original and log-transformed models.*

**Table S4. Diagnostic Assessment of Index-Component Correlation**

| **Index** | **Component** | **Spearman_Rho** | **Warning** |
| --- | --- | --- | --- |
| NLR | Neutrophil | 0.650 | Normal |
| NLR | Lymphocyte | -0.693 | Normal |
| SII | PLT | 0.453 | Normal |
| SII | Neutrophil | 0.712 | Normal |
| SII | Lymphocyte | -0.483 | Normal |
| CAR | hsCRP | 0.983 | High Collinearity (>0.8) |
| CAR | ALB | -0.251 | Normal |
| FAR | Fibrinogen | 0.910 | High Collinearity (>0.8) |
| FAR | ALB | -0.418 | Normal |
| PNI | ALB | 0.786 | Normal |
| PNI | Lymphocyte | 0.650 | Normal |

**Table S5. Collinearity Diagnostics of the Coagulation Axis**

| **Pair** | **Spearman_Rho** | **Conclusion** |
| --- | --- | --- |
| INR vs PT | 0.945 | Separate Runs Needed |
| INR vs APTT | 0.225 | Separate Runs Needed |
| PT vs APTT | 0.240 | Separate Runs Needed |

**Table S6. Univariate Logistic Regression Analysis**

| **Index** | **Direction** | **OR_Unit** | **OR_SD** | **P_Value** | **AUC_95CI** | **Cutoff** | **Sens** | **Spec** |
| --- | --- | --- | --- | --- | --- | --- | --- | --- |
| NLR | Risk | 1.15 (1.07-1.23) | 1.46 (1.20-1.78) | <0.001 | 0.632 (0.573-0.691) | 3.22 | 52.3% | 69.0% |
| SII | Risk | 1.00 (1.00-1.00) | 1.36 (1.08-1.70) | 0.008 | 0.633 (0.575-0.691) | 435.58 | 84.7% | 37.5% |
| hsCRP | Risk | 1.01 (1.00-1.02) | 1.28 (1.05-1.55) | 0.013 | 0.652 (0.596-0.708) | 1.35 | 72.1% | 52.6% |
| CAR | Risk | 1.55 (1.08-2.22) | 1.27 (1.04-1.55) | 0.017 | 0.639 (0.581-0.698) | 0.09 | 51.4% | 71.9% |
| FAR | Risk | 1.13 (1.05-1.22) | 1.38 (1.13-1.70) | 0.002 | 0.590 (0.528-0.652) | 8.00 | 66.7% | 50.3% |
| ALB | Protective | 0.95 (0.91-0.99) | 0.78 (0.63-0.97) | 0.026 | 0.569 (0.508-0.629) | 43.69 | 78.4% | 33.1% |
| HbA1c | Risk | 1.17 (1.04-1.32) | 1.31 (1.07-1.59) | 0.008 | 0.575 (0.515-0.636) | 6.05 | 65.8% | 47.1% |
| CHG | Risk | 1.48 (1.03-2.13) | 1.25 (1.01-1.53) | 0.036 | 0.562 (0.500-0.623) | 2.64 | 55.9% | 56.5% |
| PNI | Protective | 0.95 (0.92-0.99) | 0.74 (0.59-0.92) | 0.007 | 0.588 (0.527-0.648) | 51.28 | 76.6% | 40.1% |

*Note: OR_Unit represents per 1 unit change. OR_SD represents per 1 Standard Deviation change. To ensure physiological interpretability, FAR values were multiplied by 100 before calculation.*

**Table S7. Pairwise DeLong Tests for AUC Comparisons**

| **Index_A** | **AUC_A** | **Index_B** | **AUC_B** | **Z_Value** | **P_Value_Raw** | **Q_Value_BH** | **Significance** |
| --- | --- | --- | --- | --- | --- | --- | --- |
| NLR | 0.758 | ALB | 0.757 | 0.138 | 0.890 | - | NS |
| NLR | 0.758 | SII | 0.757 | 0.328 | 0.743 | 0.984 | NS |
| NLR | 0.758 | hsCRP | 0.752 | 0.781 | 0.435 | 0.913 | NS |
| NLR | 0.758 | CAR | 0.752 | 0.774 | 0.439 | 0.913 | NS |
| NLR | 0.758 | PNI | 0.757 | 0.103 | 0.918 | 0.984 | NS |
| SII | 0.757 | hsCRP | 0.752 | 0.726 | 0.468 | 0.913 | NS |
| SII | 0.757 | ALB | 0.757 | 0.020 | 0.984 | 0.984 | NS |
| SII | 0.757 | PNI | 0.757 | -0.035 | 0.972 | 0.984 | NS |
| hsCRP | 0.752 | ALB | 0.757 | -0.700 | 0.484 | 0.913 | NS |
| hsCRP | 0.752 | PNI | 0.757 | -0.720 | 0.472 | 0.913 | NS |
| CAR | 0.752 | ALB | 0.757 | -0.752 | 0.452 | 0.913 | NS |
| CAR | 0.752 | PNI | 0.757 | -0.759 | 0.448 | 0.913 | NS |
| FAR | 0.751 | ALB | 0.757 | -0.717 | 0.473 | 0.913 | NS |
| ALB | 0.757 | PNI | 0.757 | -0.120 | 0.904 | 0.984 | NS |

*Note: NS = Not Significant.*

**Table S8. Dose-Response Restricted Cubic Spline (RCS) Inference**

| **Index** | **Skewness** | **Knots Used** | **Ref Percentile** | **Ref Value** | **P for overall** | **P for nonlinearity** | **Curve Inference** |
| --- | --- | --- | --- | --- | --- | --- | --- |
| NLR | 3.55 | 4 | 25th | 1.933 | 0.008 | 0.367 | Significant Linear |
| SII | 6.77 | 5 | 25th | 384.554 | 0.009 | 0.068 | Significant Linear |
| hsCRP | 6.15 | 4 | 25th | 0.500 | 0.004 | 0.002 | Significant Non-linear (U/J-shape) |
| CAR | 6.81 | 3 | 25th | 0.013 | 0.008 | 0.004 | Significant Non-linear (U/J-shape) |
| FAR | 3.33 | 3 | 25th | 6.800 | 0.151 | 0.808 | Non-significant overall effect |
| ALB | 0.04 | 5 | 50th | 41.600 | 0.484 | 0.951 | Non-significant overall effect |
| HbA1c | 1.46 | 4 | 50th | 6.200 | 0.655 | 0.751 | Non-significant overall effect |
| CHG | 0.74 | 4 | 50th | 2.589 | 0.983 | 0.936 | Non-significant overall effect |
| PNI | 0.08 | 5 | 50th | 49.430 | 0.249 | 0.854 | Non-significant overall effect |
| SIRI | 6.99 | 4 | 25th | 0.797 | 0.013 | 0.056 | Significant Linear |

*Note: Adjusted for 11 core clinical covariates.*

**Table S9. Events Per Variable (EPV) Assessment for Modeling Feasibility**

| **Assessment Criterion** | **Event Count (N)** | **Candidate Variables (P)** | **Events Per Variable (EPV)** | **Disposition Strategy** |
| --- | --- | --- | --- | --- |
| Binary Logistic (mRS > 2) | 111 | 15 | 7.40 | - |
| Ordinal Logistic (Min Category) | 28 (mRS=4) | 15 | 1.87 | - |
| Final Modeling Feasibility | - | - | 1.87 | Not Feasible: Abort joint modeling. |

**Table S10. Bootstrap Internal Validation and Optimism-Corrected Performance**

| **Metric** | **Apparent (Baseline)** | **Optimism Bias (Mean ± SD)** | **Optimism 95% CI** | **Optimism-Corrected** |
| --- | --- | --- | --- | --- |
| Ordinal C-statistic | 0.657 | 0.023 ± 0.018 | [-0.012, 0.055] | 0.634 |
| Ordinal Brier Score | 0.7117 | -0.0139 ± 0.0141 | [-0.0440, 0.0108] | 0.7256 |
| Brier Skill Score (BSS) | 0.035 | N/A | N/A | 0.016 |
| Binary Calib Slope | 1.000 (Ref) | N/A | N/A | 0.805 [0.639, 1.030] |

*Note: Calculated based on 1,000 full-process bootstrap iterations.* *Crucial Note: The Ordinal Brier Score is computed as the sum of squared errors across all 6 outcome categories (mRS 0-5). Therefore, its theoretical maximum is 2.0, unlike binary Brier scores which typically range from 0 to 0.25.*

**Table S11. Sensitivity Analysis: Simulated ORs Adjusted for Unmeasured NIHSS Confounding**

| **Index** | **Observed OR** | **Weak (R²=0.05)** | **Moderate (R²=0.15)** | **Strong (R²=0.25)** | **Reversal under Strong?** |
| --- | --- | --- | --- | --- | --- |
| NLR | 1.41 | 1.37 (-2.4%) | 1.31 (-7.0%) | 1.25 (-11.1%) | No |
| CAR | 1.22 | 1.19 (-2.4%) | 1.14 (-7.0%) | 1.08 (-11.1%) | No |
| ALB | 0.81 | 0.83 (-2.5%) | 0.87 (-7.5%) | 0.91 (-12.5%) | No |
| PNI | 0.78 | 0.80 (-2.5%) | 0.84 (-7.5%) | 0.88 (-12.5%) | No |
